# 487. Experience with Remdesivir for Treatment of SARS-CoV-2 in Patients with Liver Cirrhosis

**DOI:** 10.1093/ofid/ofab466.686

**Published:** 2021-12-04

**Authors:** Patricia Saunders-Hao, Sumeet Jain, Bruce Hirsch, Pranisha Gautam-Goyal

**Affiliations:** 1 North Shore University Hospital, New York, New York; 2 Long Island Jewish Medical Center, New Hyde Park, New York; 3 Hoftsa Northwell School of Medicine, Manhasset, NY; 4 Zucker School of Medicine at Northwell, Manhasset, New York

## Abstract

**Background:**

Remdesivir is a nucleotide analogue antiviral that was FDA approved for the treatment of hospitalized patients with coronavirus disease 2019 (COVID-19) caused by severe acute respiratory syndrome coronavirus 2 (SARS CoV-2). Remdesivir has been associated with elevations in serum aminotransferase levels but most cases being mild to moderate and reversible upon discontinuation. Although national COVID-19 guidelines and the American Association for the Study of Liver Diseases (AASLD) currently recommend remdesivir for use in hospitalized patients requiring supplemental oxygen, data is limited using remdesivir in patients with chronic liver disease. Here, we describe our experience with remdesivir in patients with liver cirrhosis.

**Methods:**

Patients with liver cirrhosis who received remdesivir were identified either prospectively or retrospectively by primary or secondary ICD-10 codes indicating liver disease. Data collected included patient demographics, underlying cause of cirrhosis, co-morbidities, Child-Pugh score, laboratory values (serum aminotransferase levels, serum creatinine) during and following remdesivir, adverse reactions attributed to remdesivir, and mortality (in-hospital, 30-day, and 90-day).

**Results:**

A total of 4 patients with underlying liver cirrhosis completed a 5-day course of remdesivir treatment. On admission, Child-Pugh class was A for 1 patient, B for 2 patients, and C for 1 patient. Causes for cirrhosis were nonalcoholic steatohepatitis (NASH), hepatic amyloidosis, and chronic hepatitis B. There were no acute elevations in aminotransferase levels or adverse events attributed to remdesivir therapy. Mortality was high with 50% in-hospital mortality. Of the 2 other patients who survived to discharge, one was discharged to home hospice and the other was readmitted within 30 days and expired during that admission.

**Conclusion:**

Since there is limited data available using remdesivir in patients with advanced liver disease, we did not identify any safety concerns related to remdesivir in our cirrhotic patients. Mortality was high illustrating the poor outcomes of patients with advanced liver disease and COVID-19. Patients with cirrhosis should be offered remdesivir if clinically appropriate.

**Disclosures:**

**All Authors**: No reported disclosures

